# Reduced brain UCP2 expression mediated by microRNA-503 contributes to increased stroke susceptibility in the high-salt fed stroke-prone spontaneously hypertensive rat

**DOI:** 10.1038/cddis.2017.278

**Published:** 2017-06-22

**Authors:** Speranza Rubattu, Rosita Stanzione, Franca Bianchi, Maria Cotugno, Maurizio Forte, Floriana Della Ragione, Salvatore Fioriniello, Maurizio D'Esposito, Simona Marchitti, Michele Madonna, Simona Baima, Giorgio Morelli, Sebastiano Sciarretta, Luigi Sironi, Paolo Gelosa, Massimo Volpe

**Affiliations:** 1 Department of Clinical and Molecular Medicine, School of Medicine and Psychology, Sapienza University of Rome, Ospedale S. Andrea, Rome, Italy; 2 Istituto di Ricovero e Cura a Carattere Scientifico (IRCCS) Neuromed, Località Camerelle, Pozzilli, Italy; 3 Institute of Genetics and Biophysics 'A. Buzzati-Traverso', Naples, Italy; 4 Food and Nutrition Research Center (CRA-NUT), Consiglio per la Ricerca in agricoltura e l’analisi dell’economia agraria, Rome, Italy; 5 Department of Medical-Surgical Sciences and Biotechnologies, Sapienza University of Rome, Latina, Italy; 6 Department of Pharmacological and Biomolecular Sciences, University of Milan, Milan, Italy; 7 Centro Cardiologico Monzino IRCCS, Milan, Italy

## Abstract

*UCP2* maps nearby the lod score peak of *STR1*-stroke QTL in the SHRSP rat strain. We explored the potential contribution of *UCP2* to the high-salt diet (JD)-dependent increased stroke susceptibility of SHRSP. Male SHRSP, SHRSR, two reciprocal SHRSR/SHRSP-*STR1/*QTL stroke congenic lines received JD for 4 weeks to detect brain UCP2 gene/protein modulation as compared with regular diet (RD). Brains were also analyzed for NF-*κ*B protein expression, oxidative stress level and *UCP2*-targeted microRNAs expression level. Next, based on knowledge that fenofibrate and *Brassica Oleracea* (BO) stimulate *UCP2* expression through PPAR*α* activation, we monitored stroke occurrence in SHRSP receiving JD plus fenofibrate *versus* vehicle, JD plus BO juice *versus* BO juice plus PPAR*α* inhibitor. Brain *UCP2* expression was markedly reduced by JD in SHRSP and in the (SHRsr.SHRsp-(*D1Rat134-Mt1pa*)) congenic line, whereas NF-*κ*B expression and oxidative stress level increased. The opposite phenomenon was observed in the SHRSR and in the (SHRsp.SHRsr*-(D1Rat134-Mt1pa)*) reciprocal congenic line. Interestingly, the *UCP2*-targeted rno-microRNA-503 was significantly upregulated in SHRSP and decreased in SHRSR upon JD, with consistent changes in the two reciprocal congenic lines. Both fenofibrate and BO significantly decreased brain microRNA-503 level, upregulated *UCP2* expression and protected SHRSP from stroke occurrence. *In vitro* overexpression of microRNA-503 in endothelial cells suppressed *UCP2* expression and led to a significant increase of cell mortality with decreased cell viability. Brain *UCP2* downregulation is a determinant of increased stroke predisposition in high-salt-fed SHRSP. In this context, UCP2 can be modulated by both pharmacological and nutraceutical agents. The microRNA-503 significantly contributes to mediate brain *UCP2* downregulation in JD-fed SHRSP.

The SHRSP represents a suitable animal model for the investigation of the etiopathogenetic basis of hypertensive target organ damage.^1^ Feeding SHRSP with JD accelerates both renal and cerebrovascular damage occurrence^2, 3^ with renal damage preceding stroke.^2, 4, 5^ The gene encoding UCP2 maps nearby the lod score peak of *STR1*/stroke QTL identified on rat chromosome 1 in the SHRSP.^3^ UCP2 is a inner mitochondrial membrane protein that exerts an antioxidant effect in various tissues by regulating fatty acid oxidation, mitochondrial biogenesis, substrate utilization and ROS elimination,^6^ and is regulated by PPAR*α*.^7^ The latter, a member of nuclear receptor family of ligand-activated transcription factors, is known to regulate lipid and energy metabolism through the uncoupling proteins;^7^ it also exerts anti-inflammatory and antioxidant effects in many cell types, including cardiovascular cells.^8^

UCP2 downregulation associates with increased oxidative stress, atherosclerosis, vascular damage and shorter lifespan in mice.^9, 10, 11, 12^ UCP2 overexpression significantly prevented ROS production in endothelial cells and preserved endothelial function by reducing ROS levels.^13, 14^ Consistently with its ability to decrease endogenous mitochondrial ROS production and to maintain normal mitochondrial membrane potential and ATP levels, a neuroprotective effect of UCP2 has been previously described both *in vitro* and *in vivo*.^15, 16, 17, 18, 19, 20, 21^ We previously reported an age-related spontaneous decrease of UCP2 gene and protein expression only in the brain of SHRSP, preceding spontaneous stroke occurrence at 1 year of age.^22^

Of interest, we have shown that, in association with increased renal injury, JD significantly downregulates UCP2 gene and protein expression in the kidneys of SHRSP, but not of SHRSR.^23^ Consistent findings were obtained in the kidneys of SHRSR/SHRSP-derived stroke congenic lines, depending on the genetic configuration of the transferred *UCP2*.^24^ *In vitro*, *UCP2* silencing in renal mesangial cells led to increased inflammation, oxidative stress and cell mortality.^23^ Exposure of primary renal proximal tubular epithelial cells isolated from SHRSP to high-NaCl medium led to UCP2 downregulation and reduced viability, which was rescued by recombinant UCP2.^24^ Moreover, the PPAR*α*-mediated upregulation of UCP2 gene and protein expression by BO sprouts juice, administered along with JD, completely prevented renal damage occurrence in SHRSP.^25^ As expected, the selective inhibition of PPAR*α* reduced the beneficial effects of BO on the renal injury of this strain.^25^

Notably, fenofibrate, a compound that exerts renal and neuroprotection in various experimental settings through its impact on several antioxidant enzymes,^26, 27, 28^ and that is also known to stimulate PPAR*α* and UCP2 expression,^29^ promoted protection from target organ damage in SHRSP.^30^

Based on the above-mentioned observations, the aims of the present study were: (1) to assess for the first time the modulation of *UCP2* in the brain of high-salt-fed SHRSP *versus* SHRSR, as well as in two SHRSR/SHRSP-*STR1*/QTL stroke congenic lines; (2) to explore the impact of PPAR*α* and *UCP2* expression modulation by BO and fenofibrate on the stroke susceptibility of high-salt-fed SHRSP; and (3) to explain part of the mechanisms underlying brain *UCP2* downregulation upon JD in the stroke-prone strain.

## Results

### Impact of 4 weeks JD feeding on brain *UCP2* expression and related inflammatory and oxidative stress parameters in the four rat lines

**Figure 1:**
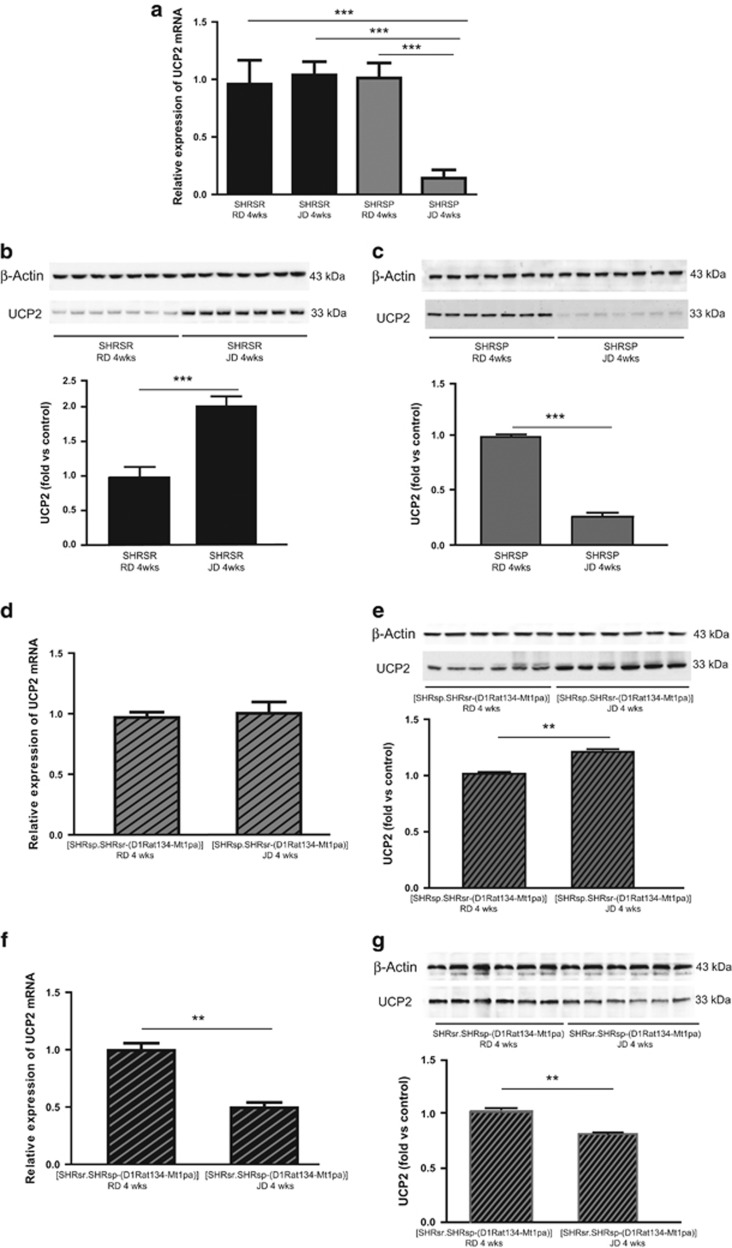
Characterization of brain UCP2 gene and protein expression upon JD in SHRSR, SHRSP and in the two SHRSR/SHRSP-*STR1*/QTL stroke congenic lines (4 weeks of dietary regimen). (**a**) *UCP2* expression in the two parental lines upon either RD or JD; *n*=7 for each line for each treatment. ***P<0.0001 for JD *versus* RD fed SHRSP, and for JD-fed SHRSP *versus* JD-fed SHRSR. (**b**) WB of UCP2 expression in RD and JD-fed SHRSR, with corresponding densitometric analysis. ***P<0.0001 for JD *versus* RD. (**c**) WB of UCP2 expression in RD and JD-fed SHRSP, with corresponding densitometric analysis. ***P<0.0001 for JD *versus* RD. (**d**) *UCP2* expression in the SHRSP-derived stroke congenic line upon RD or JD; *n*=6 for each treatment. (**e**) WB of UCP2 expression in the SHRSP-derived stroke congenic line upon RD or JD. **P<0.001 for JD *versus* RD. (**f**) *UCP2* expression in the SHRSR-derived stroke congenic line upon RD or JD. *n*=6 for each treatment. **P<0.001 for JD *versus* RD. (**g**) WB of UCP2 expression in the SHRSR-derived stroke congenic line upon RD or JD. **P<0.001 for JD *versus* RD

Four weeks of JD feeding induced a significant UCP2 gene and protein expression downregulation only in the SHRSP brain (Figures 1a–c), as previously reported in the kidneys.^23, 24, 25^ The (SHRsp.SHRsr-(D1Rat134-Mt1pa)) congenic line, derived from the SHRSP parental strain and carrying the SHRSR/*STR1* chromosomal fragment, did not downregulate *UCP2* under JD, differently from the SHRSP strain of origin (Figures 1d and e). Vice versa, the (SHRsr.SHRsp-(D1Rat134-Mt1pa)) congenic line, derived from the SHRSR parental strain and carrying the SHRSP/*STR1* chromosomal fragment, significantly downregulated *UCP2* under JD, differently from the SHRSR strain of origin (Figures 1f and g). These results confirmed the key role of *UCP2* configuration (SP or SR) for the response to high-salt diet.

**Figure 2:**
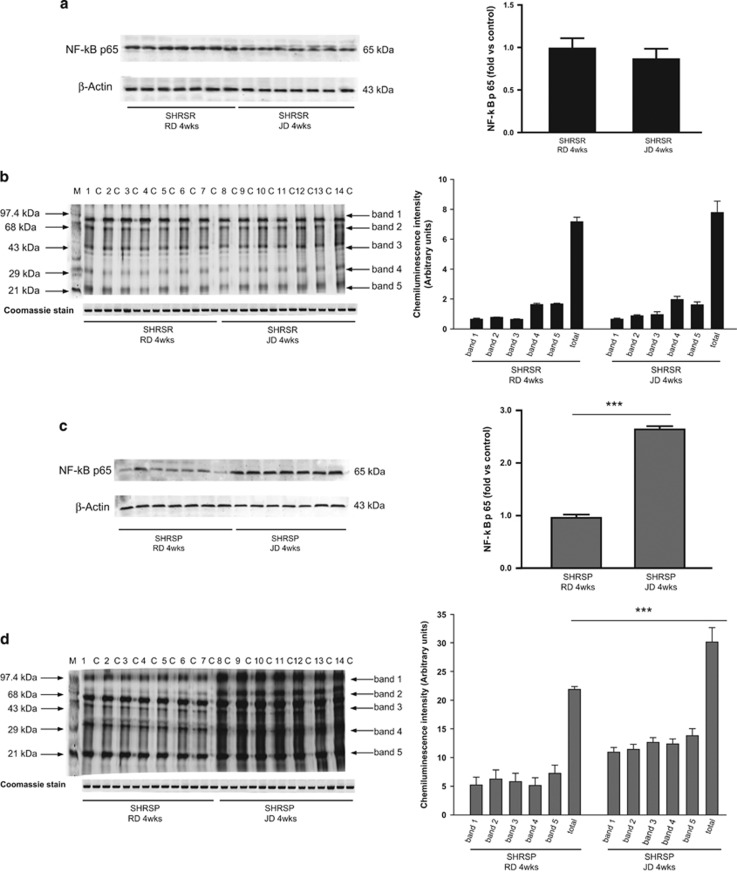
Characterization of NF-*κ*B protein expression and of oxidative stress level in brains of JD-fed SHRSR and SHRSP (4 weeks of dietary regimen). (**a**) WB of NF-*κ*B expression with corresponding densitometric analysis in SHRSR upon RD or JD. (**b**) WB of carbonylated total proteins in SHRSR upon RD or JD. Each lane was loaded with 50 *μ*g of total proteins. Lane M, DNP marker. Each sample was run with its own untreated control (C). Normalization for lane protein loading was performed using Coomassie staining. The corresponding densitometric analysis is shown on the right side of the panel. Bar graphs represent chemiluminescence intensity relative to the gel loading band. Bands 1 to 5 refer to the most prominent bands on the blots (identified by arrows), whereas total refers to the total chemiluminescence intensity from all bands. (**c**) WB of NF-*κ*B expression with corresponding densitometric analysis in SHRSP upon RD or JD. ***P<0.0001 for JD *versus* RD. (**d**) WB of carbonylated total proteins in SHRSP upon RD or JD with corresponding densitometric analysis shown on the right side of the panel. See legend of panel (**b**). ***P<0.0001 for JD *versus* RD

**Figure 3:**
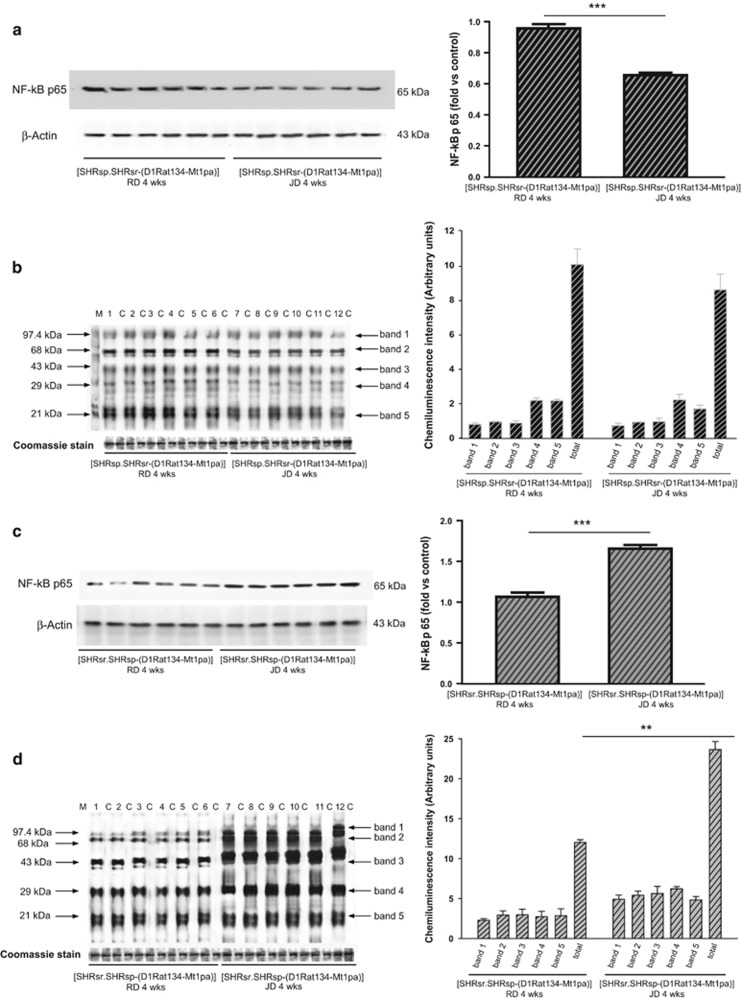
Characterization of NF-*κ*B protein expression and of oxidative stress level in brains of JD-fed SHRSR/SHRSP-*STR1*/QTL stroke congenic lines (4 weeks of dietary regimen). (**a**) WB of NF-*κ*B and (**b**) of carbonylated total proteins in the SHRSP-derived stroke congenic line upon RD or JD. See legend of Figure 2b, for the WB of carbonylated total proteins. Bar graphs on the right side represent corresponding densitometric analysis. ***P<0.0001 for JD *versus* RD. (**c**) WB of NF-*κ*B and (**d**) of carbonylated total proteins in the SHRSR-derived stroke congenic line upon RD or JD and corresponding densitometric analysis shown on the right side. See legend of Figure 2b for the WB of carbonylated total proteins. ***P<0.0001 for JD *versus* RD. (**d**) **P<0.001 for JD *versus* RD

Figure 2 shows the NF-*κ*B protein expression level, a marker of inflammation, and the carbonylated protein level, a marker of oxidative stress, in the brains of the parental lines (SHRSR: panels a and b; SHRSP: panels c and d). Both inflammatory and oxidative stress markers were significantly increased only in the brains of JD-fed SHRSP. Figure 3 shows the same parameters in the two *STR1*/QTL stroke congenic lines, the one derived from the SHRSP (panels a and b), and the one derived from the SHRSR (panels c and d). Both inflammatory and oxidative stress markers were decreased in the SHRSP-derived stroke congenic line, carrying the SHRSR/*STR1* chromosomal fragment (panels a and b), whereas these markers increased significantly in the SHRSR-derived stroke congenic line carrying the SHRSP/*STR1* chromosomal fragment (panels c and d). These results confirmed that, whenever *UCP2* expression was downregulated, such as in JD-fed SHRSP and JD-fed (SHRsr.SHRsp-(D1Rat134-Mt1pa)), the degree of inflammation and of oxidative stress increased. Vice versa, no increase of these processes was detected in the brains of both JD-fed SHRSR and JD-fed (SHRsp.SHRsr-(D1Rat134-Mt1pa)), both carrying higher levels of brain *UCP2* expression (as compared with the other two lines).

### Impact of fenofibrate administration on brain *UCP2* expression and on stroke occurrence in JD-fed SHRSP

**Figure 4:**
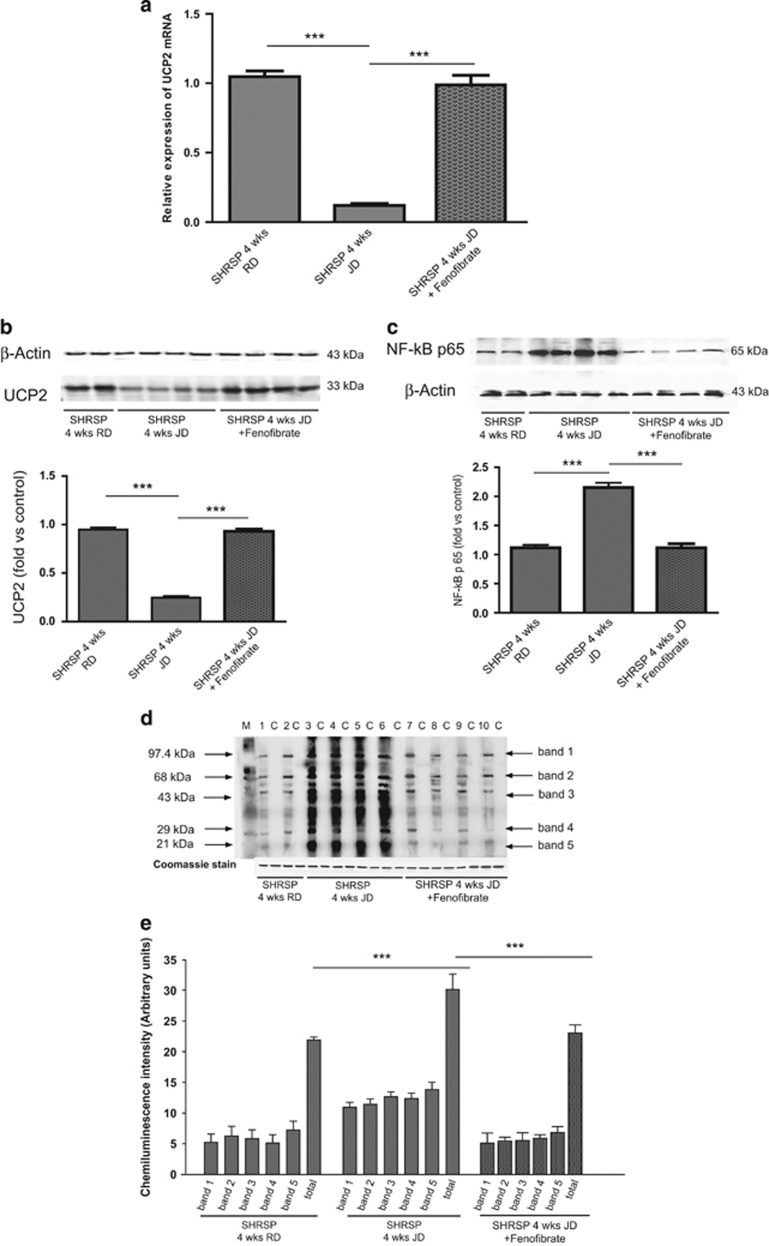
Impact of fenofibrate administration for 4 weeks on brain UCP2 modulation, NF-*κ*B protein expression, oxidative stress level in JD-fed SHRSP. (**a** and **b**) UCP2 gene and protein expression levels; (**c**) NF-*κ*B expression level; (**d** and **e**) oxidative stress level; *n*=4 for each experimental group. ***P<0.0001 for each comparison

Figure 4 shows the impact of JD plus fenofibrate *versus* JD alone on brain UCP2 gene and protein expression, and on NF-*κ*B and oxidized total protein levels at the end of 4 weeks of treatment. Fenofibrate could restore UCP2 level (panels a and b) and decrease levels of both NF-*κ*B and oxidative stress (panels c–e).

**Figure 5:**
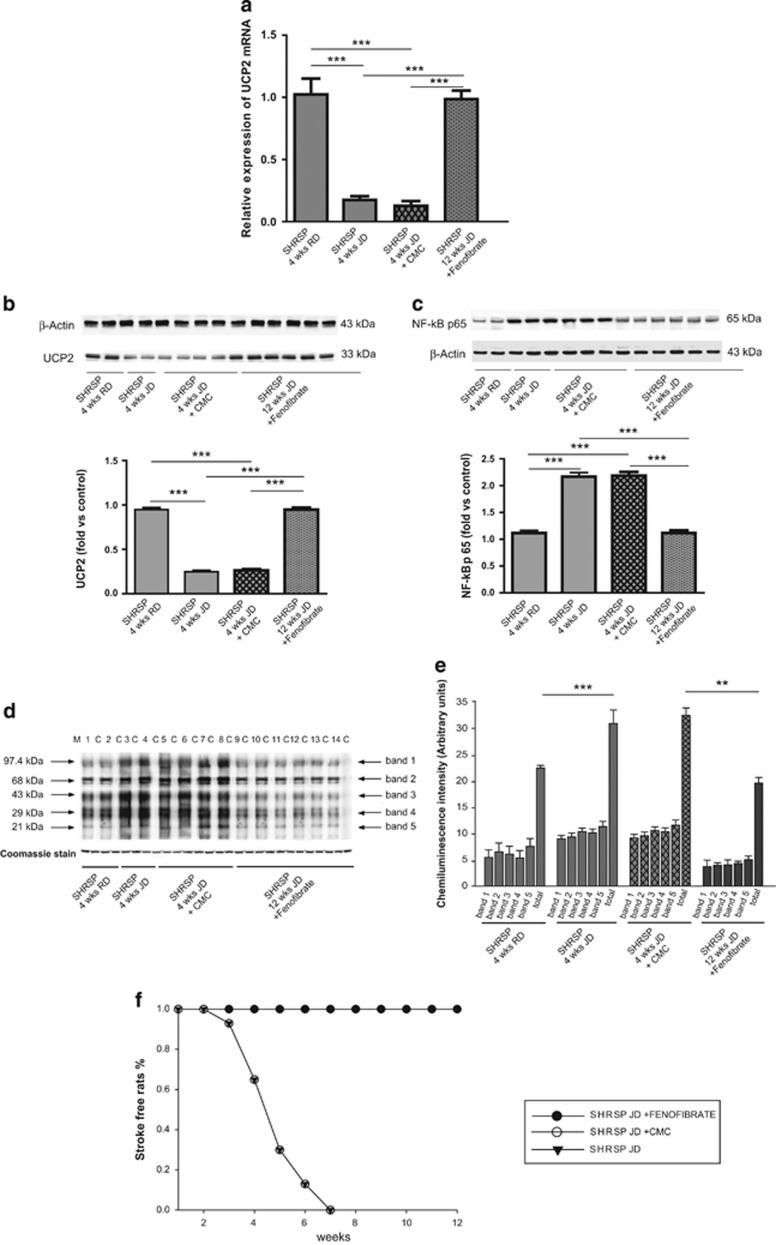
Impact of long-term fenofibrate administration on brain *UCP2* modulation, NF-*κ*B protein expression, oxidative stress and stroke occurrence in JD-fed SHRSP. (**a**) *UCP2* expression is shown in SHRSP fed for 4 weeks with RD (*n*=7), JD (*n*=7), JD plus vehicle (*n*=4) and at the end of three months of JD plus fenofibrate treatment (*n*=6). ***P<0.0001 for each comparison. (**b**) WB of UCP2 expression in the four experimental groups as above with corresponding densitometric analysis. ***P<0.0001 for each comparison. (**c**) WB of NF-*κ*B expression in the four experimental groups as above with corresponding densitometric analysis. ***P<0.0001 for each comparison. (**d**) WB of carbonylated total proteins in the four experimental groups with corresponding densitometric analysis (**e**). See legend of Figure 2b, for the WB of carbonylated total proteins. ***P<0.0001 for JD *versus* RD fed SHRSP; **P<0.001 for JD plus fenofibrate *versus* JD plus CMC fed SHRSP. (**f**) Stroke survival rate in the JD, JD plus vehicle and JD plus fenofibrate treated SHRSP. The comparison of JD plus fenofibrate treated SHRSP *versus* both JD and JD plus vehicle treated SHRSP was significant, *P*<0.001

Figure 5 shows the results of the stroke survival study performed with a long-term fenofibrate administration (3 months) in JD-fed SHRSP. The impact on UCP2 gene and protein expression levels in brains of JD plus fenofibrate treated SHRSP, as compared with animals receiving JD only and JD plus vehicle, is shown in the panels a and b of the Figure 5. Also at the end of 3 months of treatment, the parallel administration of JD and fenofibrate restored UCP2 level (panels a and b), and decreased levels of both NF-*κ*B and oxidative stress despite the long-term treatment with JD (panels c–e). Importantly, fenofibrate fully protected animals from stroke occurrence over 3 months of follow-up (panel f). In contrast, occurrence of stroke events reached 100% by the seventh week of JD in both JD and JD plus vehicle treated rats, consistently with previous evidence.^3, 5^ SBP and BW values upon fenofibrate administration are reported in the Supplementary Table S1.

### Impact of BO administration on brain *UCP2* expression and on stroke occurrence in JD-fed SHRSP

Supplementary Figure S1 shows the impact of JD plus BO *versus* JD alone on brain UCP2 gene and protein expression, NF-*κ*B and oxidized total protein levels at the end of 4 weeks of treatment. BO restored UCP2 level (panels a and b) and decreased levels of both NF-*κ*B and oxidative stress (panels c–e).

**Figure 6:**
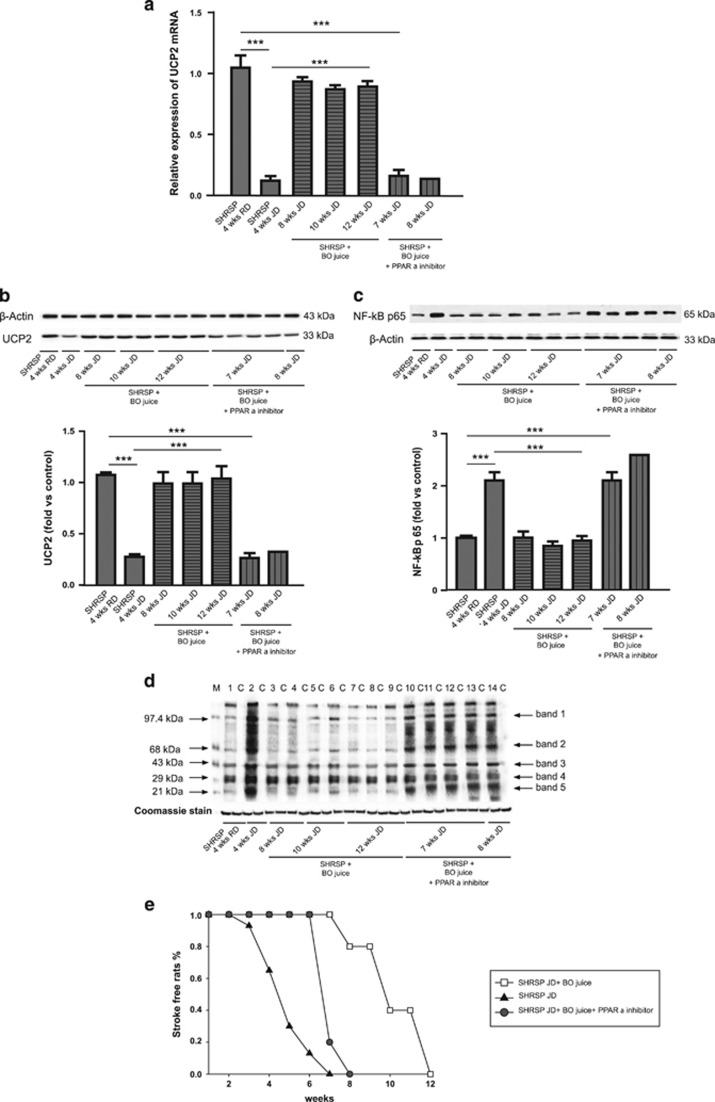
Impact of long-term administration of BO on brain *UCP2* modulation, NF-*κ*B protein expression, oxidative stress levels and on stroke occurrence in JD-fed SHRSP. (**a**) *UCP2* expression is shown in SHRSP fed for 4 weeks with RD (*n*=7), JD (*n*=7) and in SHRSP receiving both JD plus BO at times of stroke occurrence (8 weeks of treatment, *n*=2; 10 weeks, *n*=2; 12 weeks, *n*=3), SHRSP receiving JD plus BO plus PPAR*α* inhibitor (7 weeks of treatment, *n*=4; 8 weeks of treatment, *n*=1). ***P<0.0001 for each comparison. (**b**) WB of UCP2 protein expression in the four experimental groups as above with corresponding densitometric analysis. ***P<0.0001 for each comparison. (**c**) WB of NF-*κ*B protein expression in the four experimental groups as above with corresponding densitometric analysis. ***P<0.0001 for each comparison. (**d**) WB of carbonylated total proteins in the four experimental groups. See legend of Figure 2b, for the WB of carbonylated total proteins. (**e**) Stroke survival rate in the JD, JD plus BO, JD plus BO plus PPAR*α* inhibitor treated SHRSP. The comparison of JD plus BO treated SHRSP *versus* both JD and JD plus BO plus PPAR*α* inhibitor treated SHRSP was significant, *P*<0.001 and *P*<0.001, respectively

Figure 6 shows UCP2 gene and protein expression levels, at different experimental times during the stroke survival study, in brains of JD plus BO treated rats, as compared with animals receiving JD only and JD plus BO plus PPAR*α* inhibitor (panels a and b). As observed at the end of 4 weeks of the combined treatment, the concomitant administration of JD and BO restored UCP2 level, decreased levels of both NF-*κ*B and oxidative stress despite JD (Figures 6c and d and Supplementary Figure S2), and led to a significant delay of stroke occurrence (Figure 6e). In fact, 40% of rats survived until the 11th week of treatment. The PPAR*α* inhibitor significantly counteracted the stimulatory effect of the BO juice on *UCP2* expression, therefore leading to 100% stroke occurrence by the eighth week of treatment (Figures 6a–e), consistently with previous findings.^25^ The SBP and BW values upon these treatments are reported in the Supplementary Table S1.

### Analysis of *UCP2*-targeted microRNAs upon JD *versus* RD in brains of SHRSR and SHRSP

**Figure 7:**
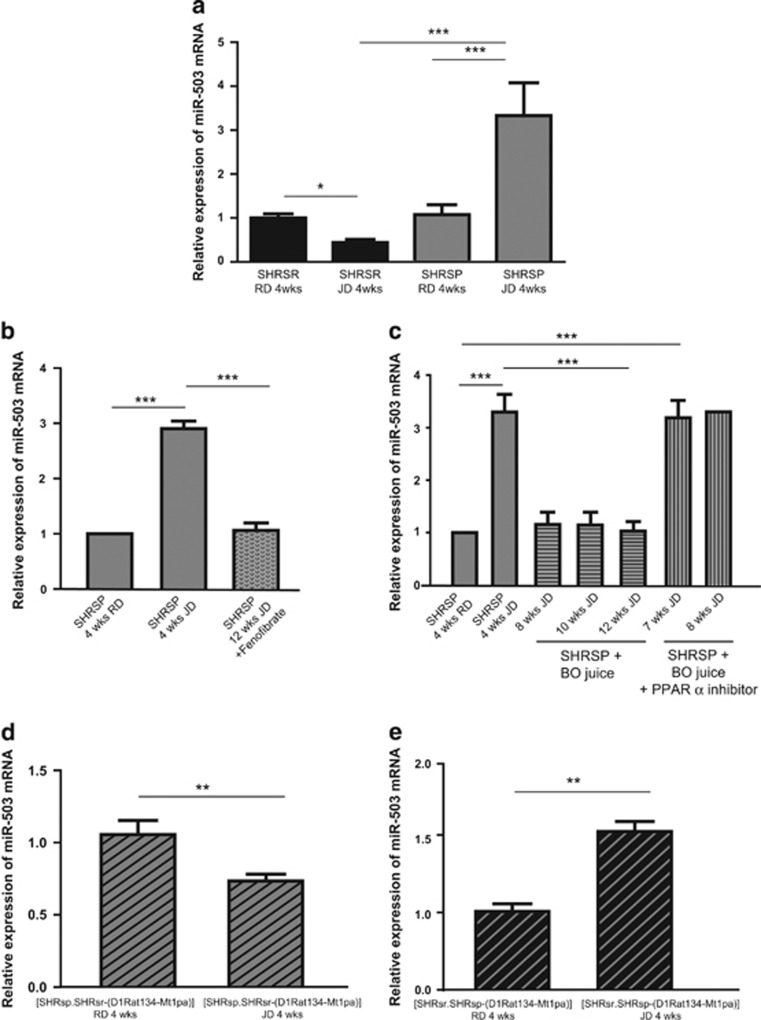
Analysis of brain rno-microRNA-503 expression level in the different experimental conditions. (**a**) miR-503 level in the SHRSR and SHRSP upon the two diets. ***P<0.0001 for JD *versus* RD fed SHRSP; ***P<0.0001 for JD-fed SHRSP *versus* JD-fed SHRSR; *P<0.05 for JD *versus* RD fed SHRSR. (**b**) Impact of fenofibrate administration for 3 months on miR-503 level in JD-fed SHRSP. ***P<0.0001 for each comparison. (**c**) Impact of BO alone and of BO plus PPAR*α* inhibitor administration on miR-503 levels in JD-fed SHRSP. ***P<0.0001 for each comparison. (**d**) miR-503 level in the SHRSP-derived stroke congenic line upon the two diets. **P<0.001 for JD *versus* RD. (**e**) miR-503 level in the SHRSR-derived stroke congenic line upon the two diets. **P<0.001 for JD *versus* RD. Rats used for this analysis were the same animals shown in previous Figures 1, 2, 3, 4, 5, 6. For number of animals see the previous figures

Out of the compared UCP2-targeted microRNAs in the brains of the SHRSR and SHRSP strains upon the two diets, we detected a remarkable differential expression, very consistent with the parallel differential *UCP2* expression, for the rno-microRNA-503. In fact, this miR was remarkably upregulated (>2 folds) in the brain of JD-fed SHRSP whereas it was significantly downregulated in the brain of JD-fed SHRSR as compared with RD (Figure 7a). No other miR showed a significant modulation in relation to the observed *UCP2* expression changes. Based on the results of the microRNAs screening, we further explored the modulation of the microRNA-503 expression in our experimental groups. We discovered that SHRSP receiving either fenofibrate or BO along with JD showed a significant reduction of brain miR-503 expression level (Figures 7b and c). The expected interference by PPAR*α* inhibitor was observed in SHRSP receiving JD and BO (Figure 7c). Furthermore, we observed a significant downregulation of brain miR-503 level in the JD-fed SHRSP-derived congenic line containing the SHRSR/*STR1* fragment (Figure 7d), whereas the SHRSR-derived congenic line, containing the SHRSP/*STR1* segment, showed a significant upregulation of miR-503 upon JD (Figure 7e). Therefore, the data obtained in the two *STR1*/QTL stroke congenic lines reinforced the evidence obtained in the parental lines of origin.

### Impact of microRNA-503 overexpression on viability of HUVECs

**Figure 8:**
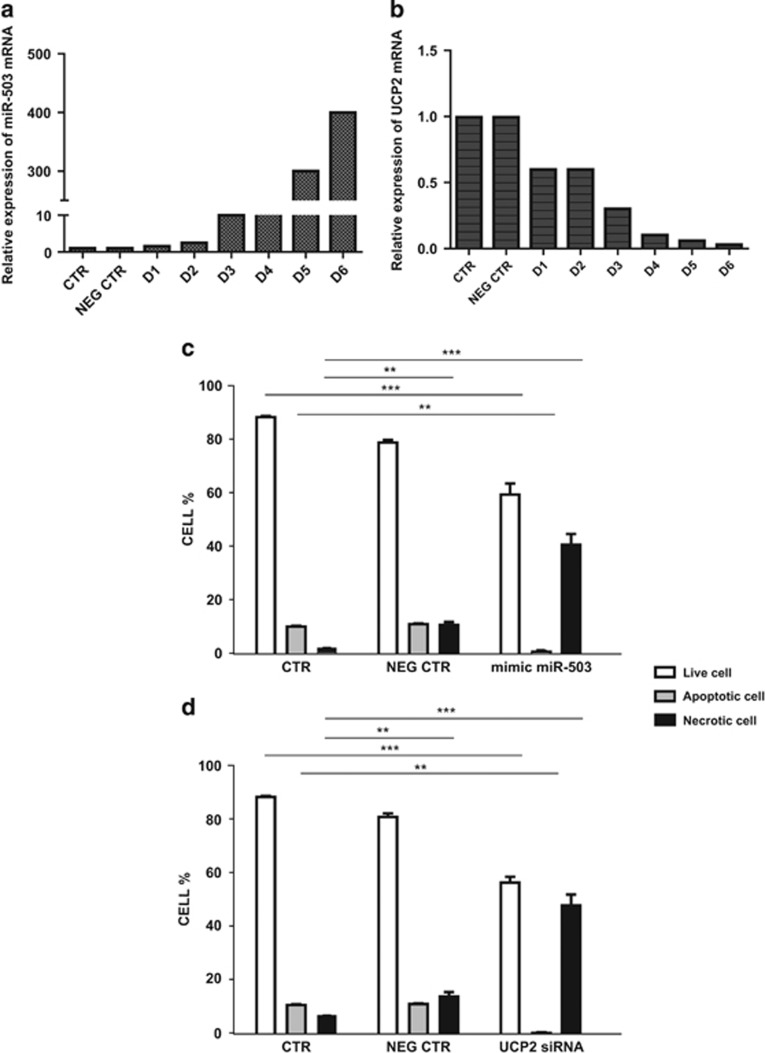
Impact of *in vitro* overexpression of hsa-microRNA-503 on *UCP2* expression and cell viability in HUVECs. (**a**) miR-503 level in HUVECs transfected with different concentrations of hsa-miR-503. (**b**) Corresponding *UCP2* expression level at each concentration of hsa-miR-503. CTR: control; NEG CTR: cells transfected with lipofectamine and mission miRNA negative control; D1: 12.5 nM; D2: 25 nM; D3: 50 nM; D4: 100 nM; D5: 200 nM; D6: 400 nM hsa-miR-503. (**c**) FACS analysis of control cells, lipofectamine-treated cells (negative control) and of cells overexpressing miRNA-503 (100 nM). ***P<0.0001 and **P<0.001 for each comparison. (**d**) FACS analysis of *UCP2* silenced cells by using a specific siRNA, compared with both control cells and negative control. ***P<0.0001 and **P<0.001 for each comparison. Each experiment was performed in triplicate. *P*=NS for comparison of UCP2 siRNA *versus* mimic hsa-microRNA-503

The *in vitro* overexpression of hsa-miR-503 in HUVECs showed a marked *UCP2* suppression with a linear dose–response (Figures 8a and b). Importantly, at a miR-503 concentration able to turn off *UCP2* expression by 90%, a significant increase of cell mortality and a significant decrease of cell viability were observed (Figure 8c). The impact on cell viability was comparable to that obtained upon direct *UCP2* silencing in HUVECs (Figure 8d).

## Discussion

Our study demonstrates that UCP2 gene and protein expression levels are significantly downregulated by Japanese style dietary feeding in brains of SHRSP but not in brains of its related control strain, the SHRSR. This phenomenon was associated with increased inflammation and oxidative stress. Accordingly, a SHRSR-derived stroke congenic line, carrying a fragment of the SHRSP-*STR1*/QTL (containing *UCP2*), showed brain *UCP2* downregulation under JD feeding associated with increased inflammation and oxidative stress. Vice versa, brain *UCP2* expression did not decrease, and both inflammation and oxidative stress were reduced upon JD in the reciprocal congenic line. Consistently, the administration of JD plus fenofibrate, known to stimulate *UCP2* expression,^25^ restored brain UCP2 levels, reduced oxidative stress and fully protected from stroke occurrence the high-salt fed SHRSP. The administration of BO sprouts extract, another stimulator of *UCP2* expression,^25^ was also able to delay significantly stroke occurrence in JD-fed SHRSP. The PPAR*α* inhibitor antagonized the beneficial effect of BO, confirming previous evidence obtained in the kidneys.^25^ The greater efficacy of fenofibrate *versus* BO on stroke protection may be explained by the additional molecular and pharmacological properties of the drug.

Of note, NF-*κ*B, which was characterized in the current study mainly for its important role in inflammation, is a ubiquitous transcription factor that, due to its wide range of gene targets, plays several other functions in mammalian cells, particularly in the nervous system.^31^

Based on our results, UCP2 appears to play an important role in the high-salt diet-dependent increased susceptibility to cerebrovascular events, as well as it does for the increased susceptibility to kidney damage of SHRSP.^23, 24, 25^ A common molecular mechanism, dependent on *UCP2* suppression, may underlie the vascular damage observed in different organs of high-salt-fed SHRSP.

Uncoupling the proton flux through UCP2 is a critical pathway in the regulation of senescence.^8, 32, 33^ The involvement of UCP2 in vascular diseases is known, being demonstrated in several pathological contexts,^9, 10, 11, 12, 13^ and it represents the consequence of its key role in the clearance of ROS within the mitochondria. A neuroprotective effect of UCP2 has been previously associated with its role on oxidative stress.^16, 17, 18, 19, 20, 21^ An association of UCP2 with stroke has been described in other experimental settings.^34, 35^

A major strength of our data relies on the evidence obtained in the reciprocal SHRSR/SHRSP-*STR1/*QTL stroke congenic lines. The stroke phenotype of these lines clearly depends on the genomic configuration of the inserted chromosomal segment belonging to *STR1*, with evidence that the chromosomal fragment carrying *UCP2* significantly interferes with stroke occurrence.^5^ Herein, we report that the introgression of the stroke-prone *STR1* chromosomal segment (carrying *UCP2)* within the stroke-resistant genomic background led to a suppression of *UCP2* expression in contrast to the upregulation of the SHRSR strain. The opposite phenomenon was observed in the reciprocal congenic line, supporting the role of the stroke-prone *UCP2* configuration to obtain downregulation in response to high-salt diet. We recently reported similar findings on UCP2 gene and protein expression with regard to renal damage in the same stroke congenic lines.^24^

A fundamental demonstration of the role of *UCP2* in stroke predisposition of SHRSP was provided by the significant protective impact of BO and fenofibrate administration, both stimulator of *UCP2* expression,^25, 26^ toward stroke occurrence despite JD feeding. Of note, our data on fenofibrate as a protective agent toward stroke confirm and extend previous findings obtained in the same animal model.^30^

No evidence of *UCP2* mutations between the two strains was obtained in our previous study.^23^ In the attempt to unravel, at least in part, some of the mechanisms underlying brain *UCP2* downregulation upon JD in the stroke-prone strain, we searched for mechanisms involved in the translational regulation of *UCP2*. As a result, we found that the *UCP2* expression modulation upon JD in the brains of SHRSP and SHRSR was related to the microRNA-503. The latter turned out to be significantly upregulated in high-salt-fed SHRSP, whereas it was significantly decreased in the SHRSR upon the same diet. Consistent results were obtained in the two congenic lines, further supporting the role of this microRNA in *UCP2* downregulation. Moreover, treatment with both fenofibrate and BO counteracted the increase of brain microRNA-503 level and the suppression of *UCP2* expression in JD-fed SHRSP. Importantly, both treatments, by their ability to restore regular levels of both microRNA-503 and UCP2, significantly protected from stroke occurrence the JD-fed SHRSP. Finally, miR-503 overexpression *in vitro* abolished *UCP2* expression and caused a high degree of cell mortality, consistently with what observed upon direct *UCP2* silencing. Our results strongly suggest that miR-503 is a modulator of brain *UCP2* expression in high-salt-fed SHRSP and also in SHRSR. Of note, miR-503 exerts multiple actions. It is reported as an antiproliferative and antiangiogenetic factor,^36^ and a cell cycle regulator; it is involved in cell adhesion, migration and angiogenesis processes.^37^ Its upregulation in diabetes strongly associates with vascular damage.^36, 38^ On the other hand, a decrease of miRNA-503 upon losartan treatment is associated with an improvement of diabetic nephropathy in an animal model of spontaneous type 2 diabetes.^39^ Herein, we report the first evidence that an increase of miR-503 associates with high-salt induced stroke occurrence, through its ability to modulate brain *UCP2* expression, in an animal model of spontaneous hypertension and stroke and that, in turn, miR-503 can be decreased by both pharmacological and nutraceutical approaches to obtain protection from stroke. Further studies will address the interaction between PPAR*α* and miR-503 in the *UCP2* regulation. It will be also interesting to characterize the potential contribution of miRNA-503 in the prevention and/or amelioration of hypertensive target organ damage with the available therapeutic antihypertensive strategies.

Our novel findings support the role of microRNAs in the end-organ damage promotion during hypertension.^40^ We are also aware, as a limitation of the current study, that the role of other still unknown *UCP2*-targeted brain microRNAs remains to be determined in our experimental conditions.

In summary, we demonstrate that *UCP2* expression downregulation by high-salt diet associates with increased stroke predisposition whereas *UCP2* upregulation, by both nutraceutical and pharmacological agents, associates with a significant stroke protection in high-salt-fed SHRSP. Our data strengthen the role of UCP2 as a suitable therapeutic target for stroke. Notably, the microRNA-503 behaves as a key determinant of the dietary-dependent regulation of *UCP2* expression in the brain of SHRSP. Thus, the microRNA-503 has a significant potential in unraveling the mechanisms underlying stroke pathogenesis and may reveal a promising therapeutic agent for this disease.

## Materials and methods

### Effects of 4 weeks JD feeding on brain UCP2 gene and protein expression in SHRSP, SHRSR and in the SHRSR/SHRSP-*STR1*/QTL stroke congenic lines

The following rat lines were used for this study: SHRSP, SHRSR, (SHRsp.SHRsr-(D1Rat134-Mt1pa)), (SHRsr.SHRsp-(D1Rat134-Mt1pa)). The latter two lines are congenic lines carrying the (D1Rat134-Mt1pa) chromosomal segment of *STR1* (containing *UCP2*) in the SHRSR configuration within the SHRSP genomic background (SHRsp.SHRsr-(D1Rat134-Mt1pa)) and, viceversa, in the SHRSP configuration within the SHRSR genomic background (SHRsr.SHRsp-(D1Rat134-Mt1pa)). The preparation of the congenic lines has been previously reported.^5^ By following our standardized experimental protocol, six-week-old male rats of both parental and congenic lines were fed with either RD or JD for 4 weeks (parental lines, *n*=7 for each line at each treatment; congenic lines, *n*=6 for each line at each treatment). At the end of 4 weeks of the dietary treatment, after SBP and BW measurement, animals were killed by cervical dislocation and brains were removed for molecular analyses. Tissue total RNA and proteins were extracted by following previously described procedures.^22, 23, 24, 25^ The UCP2 gene and protein expression levels, the NF-*κ*B protein expression level and carbonylated total proteins level were assessed by previously described procedures.^22, 23, 24, 25^

### Impact of fenofibrate administration on brain UCP2 gene and protein modulation and on stroke occurrence in JD-fed SHRSP

In order to fulfill the first aim, male SHRSP received, starting at 6 weeks of age, JD alone (*n*=4), JD plus fenofibrate (150 mg/kg/die, *n*=4) or vehicle (1% CMC, *n*=4) administered via gavage for 4 weeks. In order to analyze the impact of fenofibrate on stroke survival, 6-week-old SHRSP received JD alone (*n*=6), JD plus fenofibrate (*n*=6) or JD plus CMC (*n*=6). They were monitored for SBP, BW and stroke occurrence up to 3 months of the dietary plus fenofibrate treatment.

Brains of JD plus fenofibrate treated SHRSP, taken at the end of both 1 month and 3 months of treatment (*n*=4 and 6, respectively), were analyzed for UCP2 gene and protein expression levels, NF-*κ*B protein expression and oxidative stress levels. These molecular analyses were compared with those obtained in brains of 4-week JD-fed SHRSP from the above experimental setup and of 4-week JD plus CMC treated SHRSP.

### Impact of BO juice administration on brain UCP2 gene and protein modulation and on stroke occurrence in JD-fed SHRSP

In order to fulfill the first aim, male SHRSP received, starting at 6 weeks of age, JD alone (*n*=4) or JD plus BO sprouts extract (*n*=4) for 4 weeks by following previously reported procedures.^25^ In order to explore the impact of BO on stroke survival, male SHRSP received JD alone (*n*=6), JD plus BO (*n*=10), JD plus BO plus PPAR*α* inhibitor (*n*=6) as previously reported.^25^ SBP, BW and stroke occurrence were monitored up to 3 months of the dietary plus BO juice treatment. Brains of SHRSP, taken either at the end of 4 weeks or at the time of stroke occurrence (between the 8th and 12th week of treatment upon BO; between the 7th and 8th week of treatment upon BO plus PPARα inhibitor), were analyzed for UCP2 gene and protein expression levels, NF-*κ*B protein expression and oxidative stress levels. These molecular analyses were compared with those obtained in brains of 4-week JD-fed SHRSP from the above experimental setup.

All rats used for the experiments described in the paragraphs 1–3 were maintained at the animal facility of the Neuromed Institution in strict compliance with the guidelines set forth by the American Physiological Society. Animal protocols were approved by the Institutional Animal Care and Use Committee of the Neuromed Institution. Climate was controlled, and temperature was set at 22 °C. Diurnal 12-h cycles were kept automatically. Animals were housed two or three per cage with free access to RD (containing 22% protein, 2.7 mg/g Na^+^, 7.4 mg/g K^+^, 0.05 mg/g methionine) and tap water, unless stated otherwise. The JD contained 17.5% protein, 3.7 mg/g K^+^ and 0.03 mg/g methionine (Lab. Piccioni, Milan, Italy), and 1% NaCl was added to the drinking water.

### Analysis of *UCP2*-targeted microRNAs expression upon JD *versus* RD in brains of SHRSR and SHRSP

Based on the very limited knowledge of the rat *UCP2* brain modulation by targeted microRNAs, we selected conserved predicted UCP2-targeted microRNAs with all miRSVR scores by searching the www.microrna.org public database. The following miRNAs were considered in this study: Rno-microRNA-1, let-7a, let-7b, let-7c, let-7d, let-7i, 16, 24, 27a, 34a, 138, 206, 214, 218, 298, 497, 503. The RT-PCR for each microRNA was performed in triplicate in brain extracts of both parental strains upon the two diets by specific gene expression Taqman assays (Life Technologies). Based on the evidence of a significant microRNA-503 upregulation in the brain of JD-fed SHRSP as opposed to a significant downregulation in the brain of SHRSR (see Results section), the modulation of this miRNA was verified in JD-fed SHRSP upon fenofibrate, vehicle, BO, BO plus PPARα inhibitor administration, as well as in the brains of the two SHRSR/SHRSP-*STR1*/QTL stroke congenic lines (by analyzing the same rats used in the above described experimental groups).

### *In vitro* hsa-microRNA-503 overexpression in HUVECs

In order to verify directly the impact of microRNA-503 on *UCP2* expression levels, we performed a dose–response experiment *in vitro*. For this purpose, HUVECs (Lonza, Cambrex, Belgium) were seeded in 60-mm well plates (2 × 10^5^ cells/well) and cultured in endothelial growth medium-2 (EGM-2; Lonza) to reach a 70–80% confluence. Then, serial concentrations of 12.5, 25, 50, 100, 200 and 400 nM of hsa-microRNA-503 mimic (Mission microRNA; Sigma-Aldrich (Milan, Italy)) were incubated in OPTIMEM reduced serum medium with a nucleic acid transferring agent (lipofectamine RNAiMAX reagent (Invitrogen, Milan, Italy)) in a final volume of 2 ml/well each for 20 min. Five hours later the complex containing medium was replaced with EGM-2 medium supplemented with 10% fetal bovine serum. Cells transfected with RNAiMax lipofectamine complex and mission miRNA negative control (Sigma-Aldrich) were used as control. Twenty-four hours after transfection cells were extracted for total RNA, by the RNazol procedure,^23^ and used for the evaluation of both miR-503 and *UCP2* expression levels by RT-PCR. A specific gene expression Taqman assay (Lifetech, Waltham, MA, USA) was used to assess miR-503 levels, as reported above. The RT-PCR of *UCP2* was performed as reported above. Finally, we assessed the impact of miR-503 overexpression at 100 nM concentration (corresponding to 90% reduction of *UCP2* expression) on cell apoptosis, necrosis and viability, as assessed by FACS. The results of FACS were compared with those obtained by performing *UCP2* silencing with a specific siRNA in the same cell line (by following previously reported procedures^23^).

### Statistical analysis

All values are shown as means±S.E.M. Statistical analysis of SBP, BW, RT-PCR, WB densitometric values and FACS values was performed by one-way ANOVA followed by Bonferroni *post hoc* test. Comparisons between two groups were performed using Student's *t-*test followed by *post hoc* Mann–Whitney test. Survivor function in rats monitored over JD feeding alone, compared with JD plus the different treatments, was estimated by the life-table method. Log-rank and Wilcoxon statistics were used for testing equality of survivor functions.

Statistical significance was stated at the *P*<0.05 level. GraphPad Prism (Ver 5.01 GraphPad Software, Inc., La Jolla, CA, USA) statistical software was used for the statistical analysis.
