# Supplementary material for: Reduced brain UCP2 expression mediated by microRNA-503 contributes to increased stroke susceptibility in the high-salt fed stroke-prone spontaneously hypertensive rat
Source: Cell Death Dis. 2017 Jun 22;8(6):e2891–. doi: 10.1038/cddis.2017.278 (PMC5520932; doi:10.1038/cddis.2017.278)
Supplement: Supplementary File [file cddis2017278x1.pdf]

Supplementary file

Reduced brain UCP2 expression mediated by microRNA-503 contributes to increased stroke susceptibility in the high-salt fed stroke-prone spontaneously hypertensive rat

Speranza Rubattu[1,2], Rosita Stanzione[2], Franca Bianchi[2], Maria Cotugno[2], Maurizio Forte[2], Floriana Della Ragione[2,3], Salvatore Fioriniello[3], Maurizio D'Esposito[2,3], Simona Marchitti[2], Michele Madonna[2], Simona Baima[4], Giorgio Morelli[4], Sebastiano Sciarretta[5], Luigi Sironi[6,7], Paolo Gelosa[7], Massimo Volpe[1,2]

[1]Department of Clinical and Molecular Medicine, School of Medicine and Psychology, Sapienza University of Rome, Ospedale S.Andrea, Rome;
[2]Istituto di Ricovero e Cura a Carattere Scientifico (IRCCS) Neuromed, Località Camerelle, Pozzilli;
[3]Institute of Genetics and Biophysics "A. Buzzati-Traverso", Naples;
[4]Food and Nutrition Research Center (CRA-NUT), Consiglio per la Ricerca in agricoltura e l'analisi dell'economia agraria, Rome;
[5]Department of Medical-Surgical Sciences and Biotechnologies, Sapienza University of Rome, Latina;
[6]Department of Pharmacological and Biomolecular Sciences, University of Milan, Milan;
[7]Centro Cardiologico Monzino IRCCS, Milan;
all from Italy.

Running title: UCP2 and stroke in SHRSP

Correspondence to:
Speranza Rubattu, MD.
Clinical and Molecular Medicine Department,
School of Medicine and Psychology
Sapienza University, S.Andrea Hospital, Rome;
IRCCS Neuromed, Pozzilli (Is), Italy
e-mail: rubattu.speranza@neuromed.it
Tel. 0039 06 33775979; Fax 0039 06 33775061

Legend to Supplementary Figure 1

Impact of JD plus BO administration, as compared to JD alone, for 4 weeks on UCP2 gene and protein expression (panels A, B), NF-kB expression (panel C), oxidative stress level (panels D, E). n = 4 for each experimental group.

***P<0.0001 for each comparison.

Legend to Supplementary Figure 2

Bars represent densitometric analysis of the western blot for oxidized total proteins performed in the experiment comparing JD, JD plus BO, JD plus BO plus PPARα inhibitor treatments in JD-fed SHRSP. ***P<0.0001 for each comparison; **P<0.001 for the 4-weeks vs 12-weeks comparison.

For number of animals see legend to Figure 6.

Supplementary Figure 1

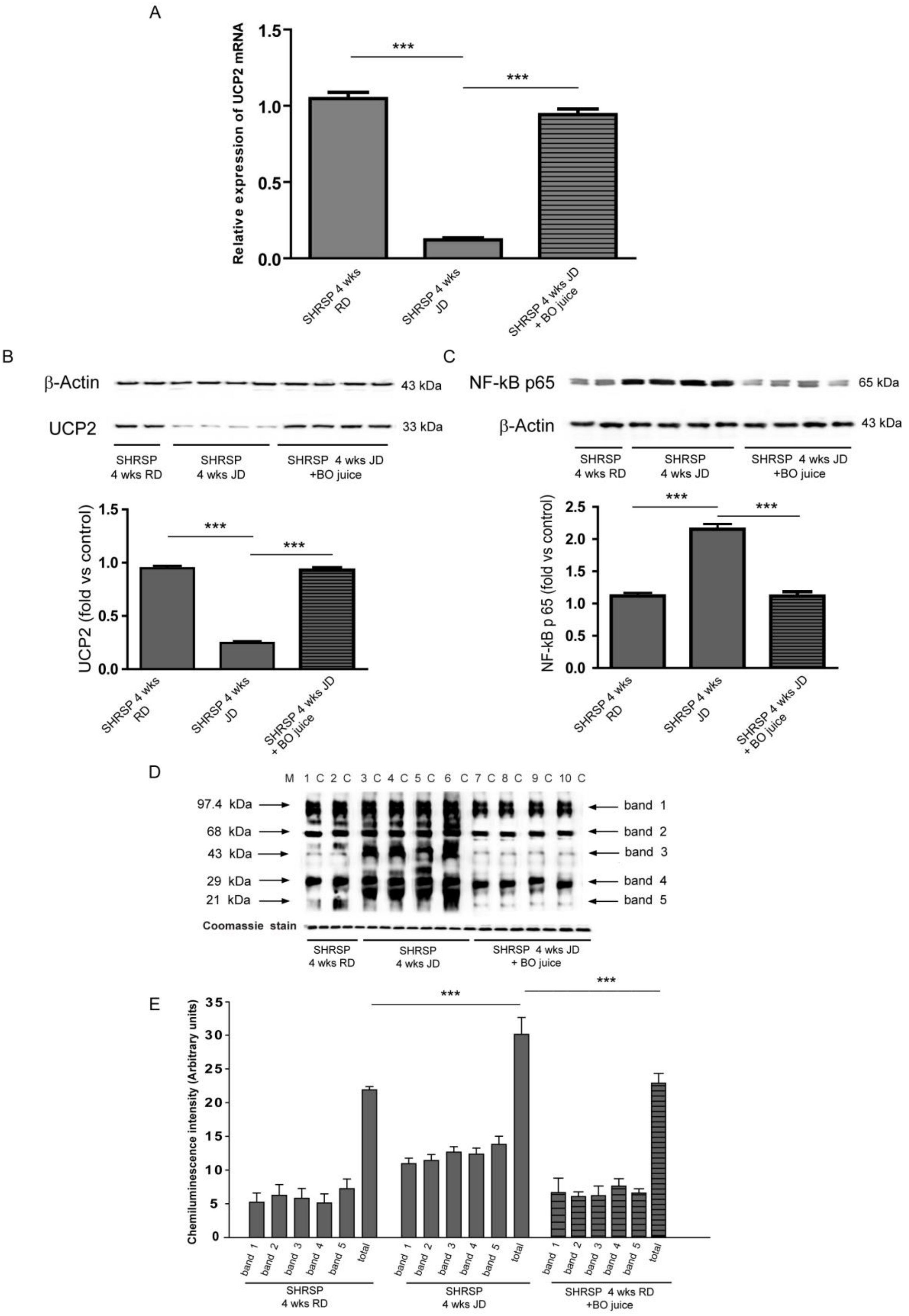

Supplementary Figure 2

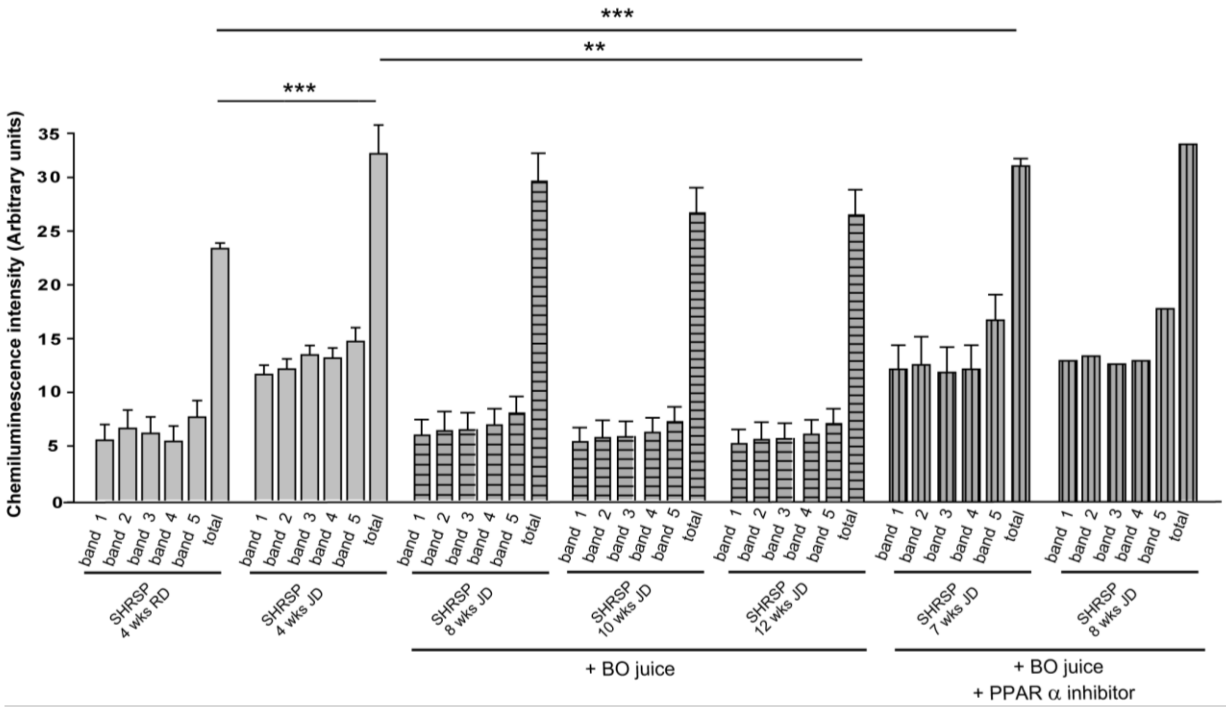

Supplementary Table 1

	JD		JD+ CMC		JD+ Fenofibrate		
**SHRSP**	**4 wks**	**6 wks**	**4 wks**	**6 wks**	**4 wks**	**8 wks**	**12 wks**
**BW (g)**	197±6	195±5	210±6	200±7	185±9	213±7 ●	229±8 ●
**SBP (mmHg)**	184±1	193±3	184±1	190±2	186±3	191±2 §	195±2 ●

	JD		JD+BO juice			JD+ BO juice + PPARα inhibitor	
**SHRSP**	**4 wks**	**6 wks**	**4 wks**	**8 wks**	**12 wks**	**4 wks**	**6 wks**
**BW (g)**	193±2	195±2	178±5	202±7 ●	225±9 ●	197±7	219±10 ♦
**SBP (mmHg)**	187±2	196±4	186±3	191±3 §	192±6 ●	184±6	192±6 ■

Legend to supplementary Table 1

BW and SBP values in the SHRSP upon JD, JD plus CMC, JD plus fenofibrate, JD plus BO, and JD plus BO plus PPARα inhibitor.

Significances are reported below for the comparisons at each treatment.

● $p<0.0001$ for 8 and 12 wks vs 4 wks JD + fenofibrate; 8 and 12 wks vs 4 wks JD + BO juice

§ $p<0.001$ for 8 wks vs 4 wks JD + fenofibrate; 8 wks vs 4 wks JD + BO juice

♦ $p<0.05$ for 6 wks vs 4 wks JD + BO juice + PPARα inhibitor

■ $p<0.001$ for 6 wks vs 4 wks JD + BO juice + PPARα inhibitor
